# Eosinophilic granulomatosis with polyangiitis following flu guard influenza vaccination: A case report

**DOI:** 10.1002/ccr3.8217

**Published:** 2023-11-25

**Authors:** Mehdi Jafarpour, Sara Daneshvar, Amir Taher Eftekharsadat, Alireza Khabbazi, Omid Pourbagherian

**Affiliations:** ^1^ Connective Tissue Diseases Research Center Tabriz University of Medical Sciences Tabriz Iran; ^2^ Department of Pathology Imam Reza Hospital Tabriz University of Medical Sciences Tabriz Iran; ^3^ Immunology Research Center Tabriz University of Medical Sciences Tabriz Iran

**Keywords:** autoimmunity, eosinophilic granulomatosis with polyangiitis (EGPA), influenza vaccine, vaccination, vasculitis

## Abstract

**Key Clinical Message:**

This case highlights a potential association between influenza vaccination and the development of eosinophilic granulomatosis with polyangiitis (EGPA), prompting the need for increased vigilance regarding vaccine‐related autoimmune reactions. While causality remains unclear, clinicians should consider this possibility in patients presenting with EGPA‐like symptoms shortly after vaccination.

**Abstract:**

Eosinophilic granulomatosis with polyangiitis (EGPA) is a rare systemic vasculitis characterized by tissue infiltration by eosinophils and hyper eosinophilia. We present a case of EGPA in a middle‐aged man following influenza vaccination. The patient developed respiratory symptoms, skin lesions, joint pain, and neurological deficits. Diagnostic tests revealed eosinophilia, positive anti‐neutrophil cytoplasmic antibodies, and elevated acute phase reactants. This report highlights a potential association between influenza vaccination and EGPA.

## Graphical abstract

Leuckocytic vasculitis with eosinophilic infiltration in dermis after influenza vaccination.

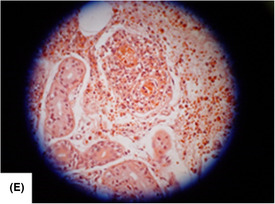

## 1 INTRODUCTION

Eosinophilic granulomatosis with polyangiitis (EGPA) or Churg‐Strauss syndrome (CSS) is a rare systemic necrotizing vasculitis affects small and medium vessels and characterized by extravascular necrotizing granulomas, tissue infiltration by eosinophils and hyper eosinophilia.^1^ Although EGPA is a multisystem disorder, the lungs, upper airways, peripheral nervous system, and skin are the most commonly involved organs.^1^ There are many reports of autoimmune disorders such as inflammatory arthritis, vasculitis, Guillain‐Barre syndrome, and multiple sclerosis after vaccination against tetanus, rubella, hepatitis B, influenza, and COVID‐19.^2^, ^3^ Here, we report a case of EGPA in a middle‐aged man after receiving the influenza vaccine.

## 2 CASE PRESENTATION

The patient is a 43‐year‐old man with a history of late‐onset asthma for the past 2 years. Before the development of EGPA symptoms, the patient's late‐onset asthma had undergone a thorough evaluation to assess potential underlying systemic diseases. However, this evaluation did not reveal any systemic diseases or significant abnormalities associated with his asthma.

**FIGURE 1:**
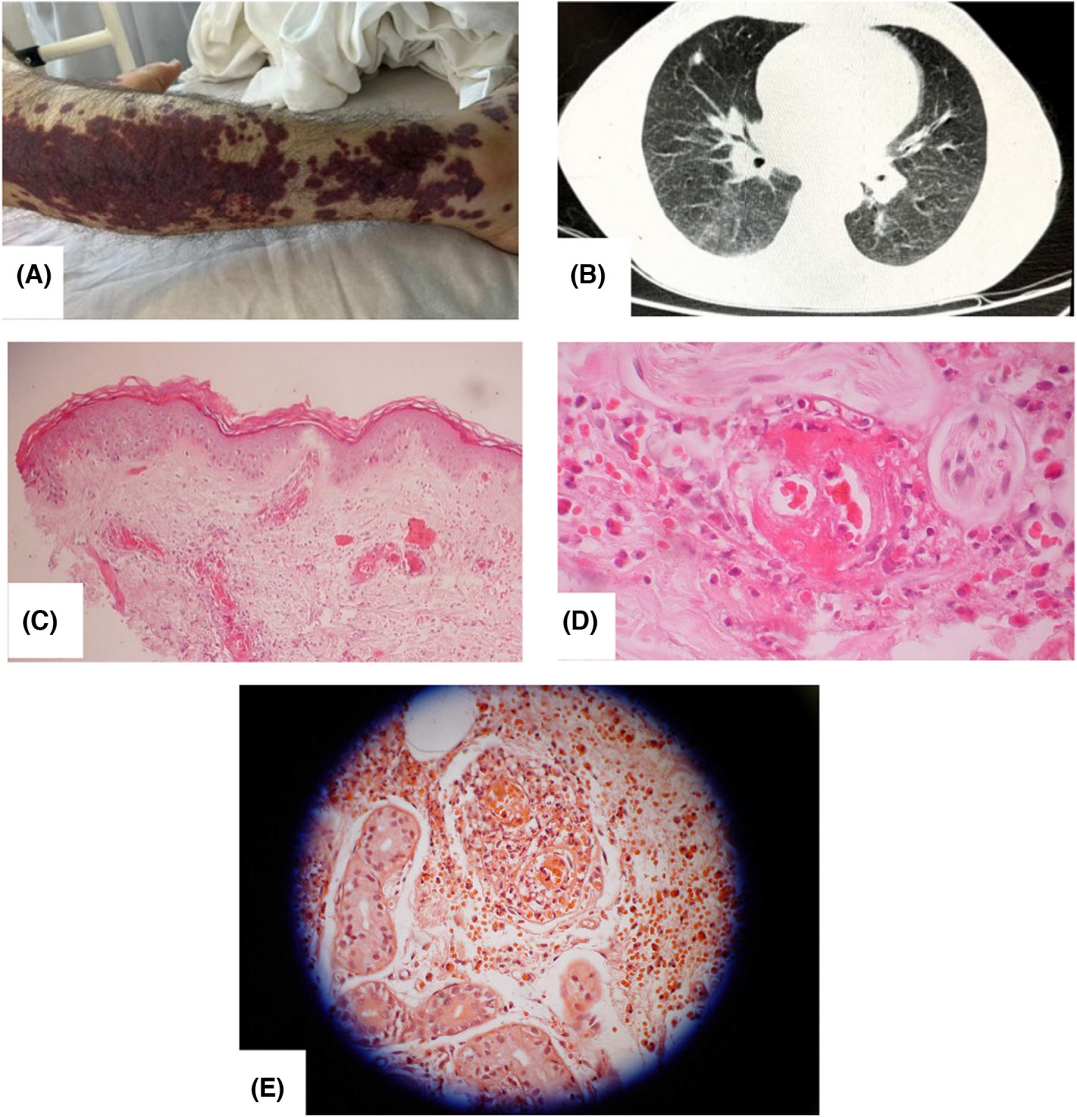
(A) Palpable petechia and purpura (patient's right lower limb); (B) Computed tomography of the chest showing mosaic pattern in the parenchyma, scattered subpleural opacities and pulmonary nodules; (C) Forearm skin biopsy (40× magnification) showing necrotic keratinocytes and subepidermal bullae; (D) Forearm skin biopsy (100× magnification) showing severe perivascular and periadnexal infiltration of lymphocytes and neutrophiles associated with nuclear debris and vascular thrombosis in the dermis and hypodermis (E) Eosinophils dyed orange‐red color with Congo red stain.

He developed shortness of breath and cough 4 days after receiving the influenza vaccine (Flu Guard). Initially, his respiratory symptoms were diagnosed as pneumonia, and he was promptly treated with antibiotics the patient was administered ceftriaxone 1 g twice daily and levofloxacin 750 mg daily. However, after a week and some improvement of the pulmonary symptoms, the patient developed skin lesions in the form of petechiae and purpura, which started from the lower limbs and gradually spread to other areas, including the face. Along with the skin lesions, the patient also complained of pain and limitation of motion of both ankles, left knee, and left elbow. His family history was negative for autoimmune diseases. The patient was admitted to the rheumatology ward. Upon examination, vital signs were found to be within normal ranges. The patient presented with notable skin abnormalities, including palpable petechiae, purpura, and hemorrhagic bullae evident across various parts of the body (Figure 1A). Furthermore, the patient reported persistent joint pain, stiffness, and swelling, particularly affecting both ankles, the left knee, and the left elbow. These clinical symptoms, including the joint abnormalities, were indicative of arthritis. On the second day of hospitalization, he experienced a tingling sensation in his upper limbs, followed by weakness in his right wrist (right wrist drop) and difficulty lifting the left foot (left foot drop). We considered a range of differential diagnoses, including other forms of vasculitis like giant cell arteritis (GCA) and Kawasaki disease, autoimmune diseases like systemic lupus erythematosus (SLE) and Sjögren's syndrome, and respiratory conditions chronic obstructive pulmonary disease (COPD), and interstitial lung disease (ILD), such as idiopathic pulmonary fibrosis (IPF), which warranted the ordering of ANCA, complements, RF, and spirometry to help differentiate and narrow down potential underlying causes.

The laboratory tests showed elevated levels of acute‐phase reactants, including erythrocyte sedimentation rate (ESR) and C‐reactive protein (CRP) along with leukocytosis, eosinophilia, and positive perinuclear anti‐neutrophil cytoplasmic antibodies (P‐ANCA) (Table 1). The EMG‐NCV results revealed a significant neurophysiological profile. Notably, all sensory nerve action potentials (SNAPs) and compound muscle action potentials (CMAPs) displayed low amplitude or were unobtainable, indicating a profound axonal motor dysfunction, surpassing sensory involvement. Furthermore, neurogenic changes were evident in several sampled muscles. These findings collectively point toward the presence of moderate to severe axonal motor‐dominant peripheral polyneuropathy. In addition, the study highlighted evidence of mononeuritis multiplex in specific nerves, further suggesting an underlying vasculitic etiology.

**TABLE 1:** Laboratory parameters of the patient.

Laboratory parameters	Patient's values	Normal range
Leukocyte count, per μL	13.64 × 10^3^ (45% Neut, 7% Lymph, 47% Eos)	4–10 × 10^3^
Hemoglobin, g/dL	15.2	12.3–15.3
Platelet count, per μL	203,000	150,000–450,000
ESR, mm/h	85	0–30
CRP, mg/L	75	< 6
AST, g/dL	33	8–35
ALT, g/dL	35	8–35
ALP, IU/L	256	100–300
BUN, mg/dL	18	7–20
Creatinine, mg/dL	1.16	0.5–1.1
Urine analysis	Protein: negative; WBC: 0–1; RBC: 0–1	
HBs Ag, IU/mL	0.1	< 1
HCV Ab, IU/mL	0.1	< 1
HIV Ab, IU/mL	0.1	< 1
Serum IgG, mg/dL	1127	800–1600
Serum IgM, mg/dL	166	40–230
Serum IgA, mg/dL	86	70–400
ANA, IU/mL	0.8	< 1
Anti‐dsDNA, IU/mL	7.8	< 16
C3, mg/dL	149	90–180
C4, mg/dL	19	10–40
CH50, mg/dL	87	51–150
P‐ANCA, IU/mL	300	0–15
C‐ANCA, IU/mL	0.5	0–15
RF, IU/mL	7	0–15

Abbreviations: ALP, Alkaline Phosphatase; ALT, Aspartate Alanine Transferase; ANA, Antinuclear Antibody; AST, Aspartate Aminotransferase; BUN, Blood Urea Nitrogen; CRP, C‐Reactive Protein; dsDNA, double‐stranded DNA; Eos, Eosinophil; ESR, Erythrocyte Sedimentation Rate; HBs Ag, Hepatitis B surface Antigen; HCV Ab, Hepatitis C Antibody; HIV Ab, Human Immunodeficiency Virus Antibody; Lymph, Lymphocyte; Neut, Neutrophil; p−/c‐ANCA, perinuclear/cytoplasmic Anti‐Neutrophil Cytoplasmic Antibodies; RF, Rheumatoid Factor.

Spirometry showed severe lower airway obstruction. Electrocardiogram and echocardiography were normal. Computed tomography of the chest showed a mosaic pattern in the parenchyma, scattered subpleural opacities, and pulmonary nodules (Figure 1B). Forearm skin lesion punch biopsy was compatible with leukocytoclastic vasculitis (Figure 1C,D). additionally, eosinophils stained orange‐red with Congo red are shown in Figure 1E. Considering the history of asthma, pulmonary involvement, asymmetric polyneuropathy, skin lesions, eosinophilia, positive P‐ANCA, and leukocytoclastic vasculitis in the skin biopsy, EGPA was diagnosed. These findings were essential in ruling out alternative diagnoses and arriving at a precise and accurate diagnosis of EGPA.

Treatment with pulse intravenous (IV) methylprednisolone 1 gr/day for three consecutive days and then oral prednisolone 1 mg/kg/d, and pulse IV cyclophosphamide 1 g per month was started. The decision to include IV cyclophosphamide was based on the patient's presentation with severe vasculitis, pulmonary involvement, and neurological symptoms, which indicated the need for more aggressive immunosuppressive therapy. Four months after discharge and receiving four pulses of cyclophosphamide, the skin lesions were completely resolved and the weakness of the limbs was significantly reduced. At the patient's last follow‐up visit which was 1 month before commencing the case report and manuscript preparation, the dose of prednisolone was reduced to 15 mg/day.

## 3 DISCUSSION

We presented a 43‐year‐old man with a history of late‐onset asthma who developed signs and symptoms that fulfilled the EGPA classification criteria after receiving the influenza vaccine. The diagnostic criteria for EGPA encompass several key elements: a history of asthma, peripheral blood eosinophilia (typically exceeding 10% of the total white blood cell count), systemic vasculitis affecting various organs or systems, histopathological evidence of vasculitis with extravascular eosinophilic infiltrates in affected tissues, neuropathy (often presenting as mono‐ or polyneuropathy), sinus abnormalities (such as nasal polyposis or sinus opacities), and the exclusion of other forms of vasculitis. To confirm an EGPA diagnosis, a patient generally needs to fulfill at least four of these criteria.^4^ The exact pathogenesis of EGPA is not well understood. However, it has been suggested that exposure to various allergens, especially microbial agents in genetically predisposed patients can lead to aberrant activation of the immune system.^1^ Vaccines contain a microbe in a weakened, live or killed state, or proteins or toxins from the microbes. Mechanisms introduced for the inducing autoimmunity after viral vaccines are molecular mimicry (antigen‐specific) and bystander activation (nonspecific). Molecular mimicry refers to the amino acid similarity of foreign antigens and host organs. It has been reported that 4% of monoclonal antibodies against the virus cross‐react with the host's self‐proteins. Bystander activation refers to the non‐specific stimulation of pre‐primed autoreactive T cells, induced by exposure to high levels of cytokines produced following vaccination. New‐onset autoimmune diseases after vaccination have been widely reported.^2^ Several case reports have reported the occurrence of EGPA following vaccination. Fu et al. reported a case of EGPA presented with fever, skin lesions, neuropathy, pulmonary infiltrates, and eosinophilia 1 week after influenza A vaccine injection.^5^ Nappi et al. reported a 63‐year‐old man who developed EGPA 1 day after receiving a booster dose of SARS‐CoV‐2 vaccine.^6^ Ibrahim et al. reported another case of EGPA about 10 days after the second COVID‐19 vaccine.^7^ Jeffs et al. showed that some influenza vaccines can stimulate ANCA production in vitro.^7^ Our case bears similarities to previous instances of vasculitis reported following influenza vaccination. A comprehensive literature review identified 65 patients who developed vasculitis after receiving the influenza vaccine, as documented in a study by Toru Watanabe.^8^ The patients predominantly included elderly individuals, with a higher prevalence among females. Vasculitides associated with post‐vaccination events encompassed large vessel vasculitis, medium vessel vasculitis, small vessel vasculitis, single organ vasculitis, vasculitis associated with systemic disease, and vasculitis associated with a probable etiology. Among these cases, ANCA‐associated vasculitis was notable, with three documented deaths and three instances of severe long‐term sequelae. While most patients achieved complete recovery or remission, the severity and potential for complications underscore the importance of vigilance regarding vaccine‐related autoimmune reactions. They proposed that viral RNA found in some influenza vaccines may stimulate TLR7 (Toll‐like receptor 7) and lead to the production of ANCA (7).

It is indeed noteworthy to consider that the influenza vaccination may have acted as a potential trigger for the development of EGPA in our patient, particularly given his history of late‐onset asthma. It is plausible that EGPA might have been initially misdiagnosed, as the clinical presentation following vaccination could have led to the initial diagnosis of pneumonia. The temporal relationship between the vaccination and the onset of EGPA symptoms, along with the absence of prior systemic disease, raises the possibility that the vaccine played a contributory role in the development of this autoimmune condition.

Genetic factors in EGPA are an evolving area of research. Although our study did not involve specific genetic testing, ongoing investigations emphasize the role of genetics in EGPA. While there is no standardized genetic test for EGPA, various genetic markers and associations have been explored in the literature.^9^, ^10^, ^11^ Our study primarily focused on clinical aspects, diagnostic criteria, and treatment outcomes. The collective body of research discussed in these articles sheds light on the intricate interplay between genetic and environmental factors in ANCA‐associated vasculitis (AAV), including granulomatosis with polyangiitis (GPA), microscopic polyangiitis (MPA), and EGPA. It highlights the significance of ANCA specificity (PR3‐ vs. MPO‐ANCA) in reclassifying these disorders. Furthermore, these studies identify distinct genetic backgrounds for different AAV clinical subtypes, such as HLA‐DP1 for GPA, HLA‐DQ for MPA, and HLA‐DRB4 for EGPA, alongside non‐MHC genetic variations, which may serve as potential biomarkers for diagnosis and targeted therapies, collectively advancing our understanding of AAV's etiology.^12^, ^13^

Although, there is insufficient evidence to suggest causality, the short time between vaccination and the onset of symptoms in our patient and other reported cases could suggest the possibility of an association between the influenza vaccine and EGPA.

## 4 CONCLUSION

This case underscores the possibility that the influenza vaccination may have triggered an outbreak of EGPA, raising concerns about potential vaccine‐induced autoimmune reactions and maybe in individuals with specific genetic predispositions. While the exact mechanisms remain unclear, this 43‐year‐old man with late‐onset asthma developing EGPA symptoms following vaccination, along with other similar cases, suggests a link between vaccination and EGPA development, emphasizing the need for heightened awareness, vigilance, and further research in recognizing and managing such adverse events promptly.

## AUTHOR CONTRIBUTIONS

**Mehdi Jafarpour:** Conceptualization; data curation; investigation; resources; software; validation; writing – original draft. **Sara Daneshvar:** Resources; software; validation; writing – original draft; writing – review and editing. **Amir Taher Eftekharsadat:** Data curation; formal analysis; validation. **Alireza Khabbazi:** Formal analysis; investigation; project administration; supervision. **omid pourbagherian:** Data curation; formal analysis; investigation; resources; visualization.

## FUNDING INFORMATION

None.

## CONFLICT OF INTEREST STATEMENT

The authors declared that they have no conflict of interest.

## ETHICS STATEMENT AND CONSENT TO PARTICIPATE

Written consent was provided by the patient.

## CONSENT

Written informed consent was obtained from the patient for publication of this case report and any accompanying images. A copy of the written consent is available for review by the Editor‐in‐Chief of this journal.

## Data Availability

Data are available.
